# dbscATAC: a resource of single-cell super-enhancers/enhancers and gene markers derived from scATAC-seq data

**DOI:** 10.1093/bioinformatics/btaf364

**Published:** 2025-06-23

**Authors:** Yingmei Li, Shahid Ullah, Yumei Xian, Yazhou Sun, Zilong Zheng, Xiaoyu Ma, Ming Shi, Changlin Zhang, Tian Li, Leli Zeng, Jie Chen, Yubin Y B Deng, Fuxin Wei, Tianshun Gao

**Affiliations:** Big Data Center, The Seventh Affiliated Hospital of Sun Yat-sen University, Shenzhen 518107, China; Department of Pharmacy, The Seventh Affiliated Hospital of Sun Yat-sen University, Shenzhen 518107, China; S-Khan Lab, Mardan, Khyber Pakhtunkhwa, Takhtbhai, KP 23200, Pakistan; Big Data Center, The Seventh Affiliated Hospital of Sun Yat-sen University, Shenzhen 518107, China; Scientific Research Center, The Seventh Affiliated Hospital of Sun Yat-sen University, Shenzhen 518107, China; Big Data Center, The Seventh Affiliated Hospital of Sun Yat-sen University, Shenzhen 518107, China; Scientific Research Center, The Seventh Affiliated Hospital of Sun Yat-sen University, Shenzhen 518107, China; Big Data Center, The Seventh Affiliated Hospital of Sun Yat-sen University, Shenzhen 518107, China; Big Data Center, The Seventh Affiliated Hospital of Sun Yat-sen University, Shenzhen 518107, China; Scientific Research Center, The Seventh Affiliated Hospital of Sun Yat-sen University, Shenzhen 518107, China; Scientific Research Center, The Seventh Affiliated Hospital of Sun Yat-sen University, Shenzhen 518107, China; Department of Gynecology, The Seventh Affiliated Hospital of Sun Yat-sen University, Shenzhen 518107, China; Department of Gynecology, The Seventh Affiliated Hospital of Sun Yat-sen University, Shenzhen 518107, China; Scientific Research Center, The Seventh Affiliated Hospital of Sun Yat-sen University, Shenzhen 518107, China; Digestive Diseases Center, Scientific Research Center, The Biobank, The Seventh Affiliated Hospital of Sun Yat-Sen University, Shenzhen 518107, China; Department of General Surgery, Shanghai Children’s Medical Center, Shanghai Jiao Tong School of Medicine, Shanghai 200127, China; Scientific Research Center, The Seventh Affiliated Hospital of Sun Yat-sen University, Shenzhen 518107, China; Department of Orthopedic Surgery, The Seventh Affiliated Hospital of Sun Yat-sen University, Shenzhen 518107, China; Shenzhen Key Laboratory of Bone Tissue Repair and Translational Research, The Seventh Affiliated Hospital of Sun Yat-sen University, Shenzhen 518107, China; Big Data Center, The Seventh Affiliated Hospital of Sun Yat-sen University, Shenzhen 518107, China; Scientific Research Center, The Seventh Affiliated Hospital of Sun Yat-sen University, Shenzhen 518107, China; Shenzhen Key Laboratory of Bone Tissue Repair and Translational Research, The Seventh Affiliated Hospital of Sun Yat-sen University, Shenzhen 518107, China

## Abstract

**Motivation:**

scATAC-seq enables high-resolution mapping of cis-regulatory elements. It has been widely applied to uncover cell-type-specific regulatory networks and complement scRNA-seq analysis in numerous studies. However, a large number of datasets generated by scATAC-seq remain underutilized due to limited exploration of super-enhancers/typical enhancers and gene markers. A comprehensive resource enabling cell-type-specific annotation of cis-regulatory elements and their dynamic enhancer–gene linkages remains an urgent unmet need for scATAC-seq.

**Results:**

We present dbscATAC, a specialized single-cell database for annotating super-enhancers, gene markers, and enhancer–gene interactions derived from scATAC-seq data. Using improved machine learning algorithms, we identified 213 835 super-enhancers across 520 tissue/cell types from three species, as well as 347 484 gene markers, 13 470 526 enhancers, and 10 402 346 enhancer–gene interactions derived from 1 668 076 single cells spanning 1028 tissue/cell types in 13 species. An easy-to-use online platform with multiple analytic modules and hierarchical query options was developed for searching, browsing and visualizing single-cell super-enhancers, enhancers, and gene markers. dbscATAC provides a comprehensive resource to facilitate the exploration of enhancer landscapes, gene regulation, and cell-type-specific characteristics in single-cell epigenomics.

**Availability and implementation:**

The database with all the super-enhancer/enhancer annotation data is available at http://singlecelldb.com/dbscATAC/index.php. And the source code of dbscATAC for prediction of SEs, enhancers, and gene markers are available at https://github.com/EvansGao/dbscATAC. The source code, tissue/cell type description, and data summary can be downloaded at DOI: 10.6084/m9.figshare.28706414.

scATAC-seq, Database, Super-enhancers/enhancers, Gene markers

## 1 Introduction

The super-enhancers (SEs) are recently characterized as clusters of functionally distinct consecutive regulatory elements that include classical enhancers as well as facilitators responsible for recruiting mediator complexes to up-regulate target genes (Blayney *et al.* 2023). They interact with master transcription factors (e.g. OCT4, SOX2, and NANOG) and mediators (e.g. MED1) to form functional complexes that drive the exceptionally high expression of genes critical for mammalian cell identity and the progression of various diseases (Hnisz *et al.* 2013, Whyte *et al.* 2013, Gartlgruber *et al.* 2021). Notably, specific inhibitors (e.g. BRD4 inhibitor) targeting the SE complex have been developed and demonstrated significant efficacy in suppressing tumor growth and proliferation, underscoring the potential of SEs as therapeutic targets for cancer treatment (Jia *et al.* 2019, Thandapani 2019, Tang *et al.* 2020). These findings highlight the pivotal roles of SEs in disease mechanisms and drug discovery research.

To date, several resources, including SEA 3.0, SEdb 2.0, SEA, SEdb, dbSUPER, and SELER (Khan and Zhang 2016, Wei *et al.* 2016, Guo *et al.* 2019, Jiang *et al.* 2019, Chen *et al.* 2020, Wang *et al.* 2023), were developed to annotate SEs across various species using the ROSE (Rank Ordering of Super-Enhancers) algorithm applied to different chromatin immunoprecipitation and sequencing (ChIP-seq) data types (Loven *et al.* 2013). They employed ChIP-seq signals of key markers (e.g. H3K27ac, P300, MED1, and BRD4) as important signatures to identify SEs across diverse tissue/cell types: (i) H3K27ac is the most widely used epigenetic marker for annotating both SEs and enhancers (Gao *et al.* 2016, Khan and Zhang 2016, Chen *et al.* 2020, Gao and Qian 2020, Wang *et al.* 2023), as its binding sites exhibit significant enrichment in active enhancers (Creyghton *et al.* 2010). (ii) P300, a histone acetyltransferase, could form a complex with CBP and dynamically activates both enhancers and SEs via catalyzing the acetylation of the histones (Visel *et al.* 2009, Witte *et al.* 2015, Narita *et al.* 2021). (iii) MED1 and BRD4 are the SE-enriched transcriptional coactivators that form the condensate of nuclear puncta at SEs to relate gene control with phase separation (Sabari *et al.* 2018). While these approaches have proven effective for annotating SEs at the bulk level, none of them is able to resolve SEs at the single-cell level. Existing methods only provide insights into average SE activity across bulk tissue samples and fail to capture the internal heterogeneity of SEs within tissues. Since enhancers exhibit cell-type specificity, single-cell-based SE identification is crucial for revealing the enhancer-driven gene regulatory mechanisms specific to sub-populations (Przytycki and Pollard 2021). Therefore, a resource dedicated to annotating single-cell SEs is highly desirable.

The single-cell Assay for Transposase-Accessible Chromatin using sequencing (scATAC-seq) has been extensively utilized to profile chromatin accessibility and annotate cell-type-specific enhancers at single-cell resolution (Cao *et al.* 2018, Cusanovich *et al.* 2018, Pliner *et al.* 2018, Satpathy *et al.* 2019, Domcke *et al.* 2020, Granja *et al.* 2021, Zhang *et al.* 2021, Gao *et al.* 2022, Riegel *et al.* 2023). Studies have confirmed that millions of candidate cis-regulatory elements identified by scATAC-seq in both adult and fetal human cell types exhibit strong cell-type specificity and show significant overlap with transgenic reporter-validated active enhancers (Visel *et al.* 2007, Domcke *et al.* 2020, Zhang *et al.* 2021). Statistical analyses of ATAC-seq peak annotations have revealed that over 50% of ATAC-seq peaks correspond to enhancers (Yan *et al.* 2020). Furthermore, scATAC-seq can be integrated with single-cell RNA sequencing (scRNA-seq) to characterize functional or disease-specific enhancers and construct enhancer-driven gene regulatory networks on a large scale (Bravo González-Blas et al. 2019, Shu *et al.* 2022, Mitra *et al.* 2024). Notably, ATAC-seq was even employed to identify cell-type-specific SEs at both bulk and single-cell levels (Lee *et al.* 2021, Yates *et al.* 2021, Riegel *et al.* 2023). These findings highlight the potential of scATAC-seq as a powerful sequencing technology for annotating single-cell enhancers and SEs, providing critical insights into cell-type-specific gene regulation and disease mechanisms.

**Figure 1.**
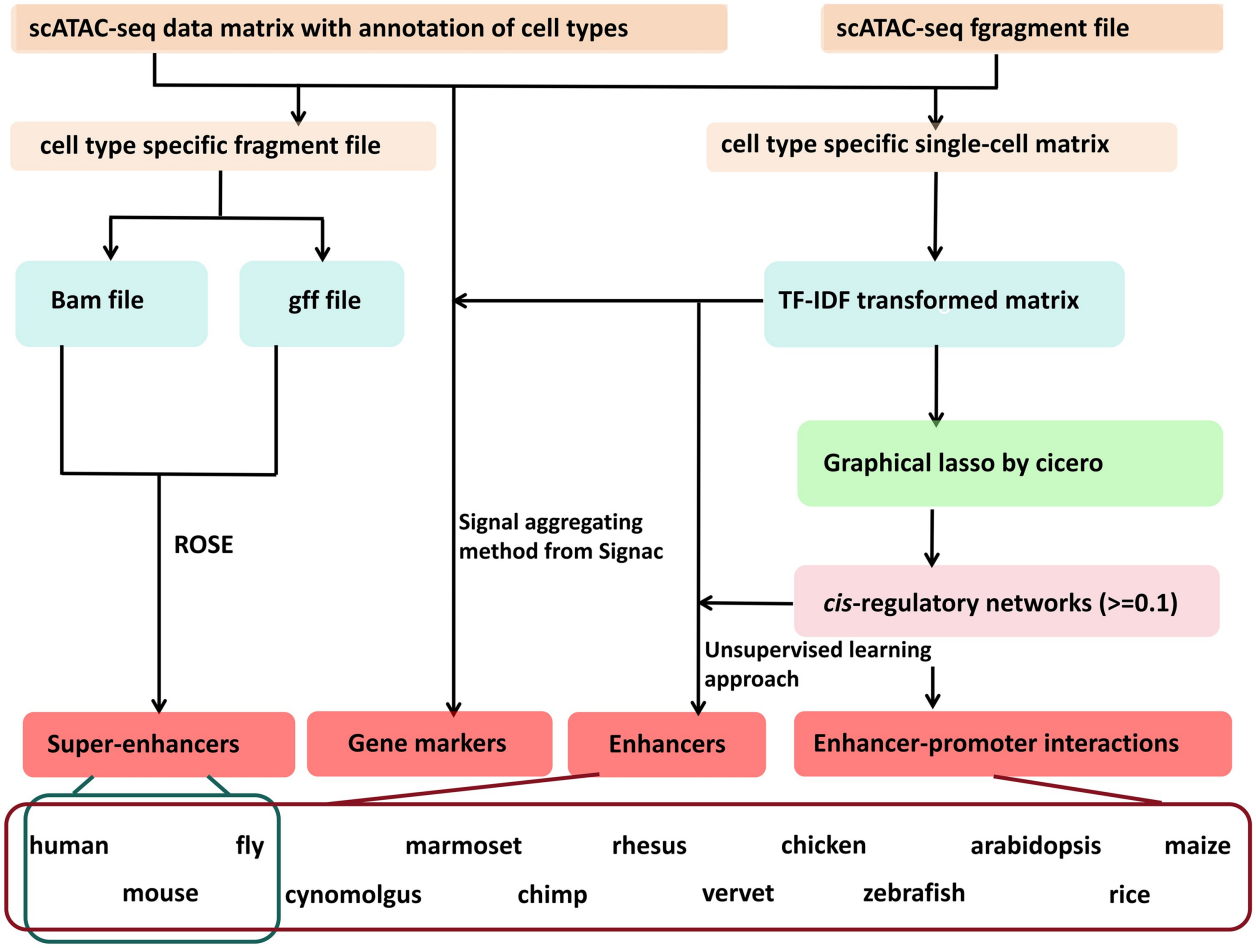
Overview of the dbscATAC pipeline.

We present dbscATAC, a comprehensive single-cell resource that utilizes scATAC-seq data to annotate tissue/cell-type-specific SEs, enhancers, and gene markers. This resource integrates multiple advanced methods, including an improved unsupervised learning approach, the ROSE algorithm, latent semantic analysis, and the graphical LASSO algorithm, to identify SEs, enhancers, and their associated genes at single-cell resolution. Compared with our previously developed enhancer databases such as EnhancerAtlas, EnhancerAtlas 2.0, and scEnhancer (Gao *et al.* 2016, Gao and Qian 2020, Gao *et al.* 2022), dbscATAC displayed five significant improvements (Fig. 1, available as supplementary data at *Bioinformatics* online): (i) Large-scale single-cell SE identification: For the first time, the ROSE algorithm was employed for large-scale identification of single-cell SEs and their associated genes in three common species. dbscATAC integrates data from 1 668 076 single cells, predicting a total of 213 835 SEs across 106, 125, and 289 tissue/cell types in human, mouse, and fly, respectively. (ii) Enhanced gene marker annotation: Latent semantic analysis and a peak-to-gene transformation method summing the peak signals within gene body and promoter regions were widely applied to annotate tissue/cell-type-specific gene markers. The methods, based on the Signac (Stuart *et al.* 2021) package, significantly improved the accuracy of gene marker identification. (iii) Improved typical enhancer and target gene identification: An enhanced unsupervised learning approach, coupled with the Cicero (Pliner *et al.* 2018), was designed to analyze hundreds of single-cell profiles, enabling the precise identification of typical enhancers and their target genes at single-cell resolution. (iv) Expanded species coverage: dbscATAC expands single-cell enhancer annotations from three species (human, mouse, and fly) to 13 species, providing a broader and more comprehensive resource for comparative analysis. (v) User-friendly web platform: A user-friendly web server with multiple modules and hierarchical browsing options were developed, allowing users to efficiently retrieve tissue/cell-type-specific SEs, gene markers, and enhancers. dbscATAC will be continuously updated with new scATAC-seq datasets and serve as a valuable resource for functional analysis of SEs at single-cell resolution, offering critical insights into gene regulation and tissue-specific enhancer activity.

## 2 Materials and methods

### 2.1 scATAC-seq data source and collection

The scATAC-seq samples of good quality were retrieved with the keyword combinations like “Single-cell ATAC-seq,” “scATAC-seq,” and “Single-cell chromatin accessibility” from various public data sources, including GEO datasets, Descartes (Domcke *et al.* 2020), 10X Genomics datasets annotated by Signac (Stuart *et al.* 2021), Mouse Atlas (Cusanovich *et al.* 2018), and Fly Brain (Taskiran *et al.* 2024). These samples span 13 species containing human (hg38), chimp (panTro5), rhesus (rheMac10), cynomolgus (macFas6), vervet (ChlSab1.1), marmoset (calJac3), mouse (mm10), chicken (galGal6), zebrafish (danRer10), fly (dm6), rice (IRGSP1), arabidopsis (TAIR10), and maize (B73v4). To standardize genome coordinates, the tool liftOver was used to convert the sample coordinates from their original assemblies to the corresponding standard reference assemblies (Hinrichs *et al.* 2006).

### 2.2 Preparation of tissue/cell-type-specific standard matrix

To obtain tissue/cell-type-specific enhancers at the single-cell level, we prepared tissue/cell-type-specific standard matrix integrated from each scATAC-seq project for calling enhancers. In the collected projects, the initial scATAC-seq data files were not uniformly formatted and were typically stored in one of several formats, including mtx, h5, Seurat RDS (Hao *et al.* 2024), cisTopic RDS (Bravo González-Blas et al. 2019), txt, ArchR RangedSummarizedExperiment (Granja *et al.* 2021) and even fastq. To standardize these data formats, we employed various R packages, including Signac, Seurat, ArchR, and cisTopic (Bravo González-Blas et al. 2019, Granja *et al.* 2021, Stuart *et al.* 2021, Hao *et al.* 2024), as well as cellranger-atac (v1.2.0) (Satpathy *et al.* 2019) to transform all initial scATAC-seq data into a unified large standard matrix format, enabling the next clustering analysis and extraction of tissue/cell-type-specific sub-populations (Table 1, available as supplementary data at *Bioinformatics* online). The large standard matrix was stored as a Seurat object, with each single cell assigned an independent bar-code and annotated with its corresponding tissue/cell type. During preprocessing, irregular cells with <200 peaks and peaks with <10 cells or located in uncommon chromosomes (e.g. chrMT) were excluded. Before normalization, peaks within the matrix were standardized to specific genome versions for each species. For example, peaks in human, chimpanzee, rhesus macaque, cynomolgus monkey, marmoset, boar, rat, mouse, chicken, zebrafish, fly, nematode, rice, Arabidopsis, and maize were corrected to the genome assemblies hg38, panTro5, rheMac10, macFas6, calJac3, Sscrofa11, rn6, mm10, galGal6, danRer10, dm6, ce10, Nip-BRI, TAIR10, and B73v4, respectively, using the liftOver tool (Hinrichs *et al.* 2006), if the original data were not already aligned to these versions.

**Table 1.** Statistical summary of single-cell tissue/cell types, SEs, gene markers, enhancers, promoters, enhancer–promoter interactions, single cells, and TF binding sites across all 13 species.

	Tissue/cell types	SEs	Gene markers	Enhancers	Promoters	Enhancer–promoter interactions	Single cells	TF binding sites
*Homo sapiens*	288	82 952	87 286	4 897 170	1 020 738	4 972 362	1 013 835	27 454 926
*Mus musculus*	178	52 373	80 958	2 327 666	533 710	3 067 750	232 257	13 585 965
*Drosophila melanogaster*	289	78 510	76 780	2 361 383	269 035	1 125 334	116 865	1 728 421
*Pan troglodytes*	26	0	16 604	1 166 288	5397	57 766	18 543	0
*Macaca mulatta*	25	0	25 628	1 079 015	11 078	88 522	11 510	0
*Macaca fascicularis*	71	0	16 148	304 242	75 390	255 704	81 475	0
*Chlorocebus sabaeus*	38	0	2165	203 589	47 494	137 924	51 728	0
*Callithrix jacchus*	39	0	12 965	683 920	57 332	191 524	23 042	0
*Gallus*	1	0	0	22 615	2003	6964	1259	0
*Danio rerio*	20	0	1814	136 781	27 988	110 504	12 354	212 737
*Arabidopsis thaliana*	19	0	16 873	20 983	7678	22 028	10 284	220 736
*Oryza sativa*	24	0	9769	99 162	22 734	61 674	38 349	0
*Zea mays*	10	0	494	167 712	53 927	304 290	56 575	0
Total	1028	213 835	347 484	13 470 526	2 134 504	10 402 346	1 668 076	43 202 785

Based on the tissue/cell type annotations assigned to all cells, the large matrix was divided into tissue/cell-type-specific sub-populations. A recent study demonstrated that binarizing the scATAC-seq matrix may obscure quantitative information and does not enhance clustering, cell type annotation, or batch correction (Martens *et al.* 2024). Therefore, we retained the scATAC-seq matrix in its original count-based format and performed analyses using a count-based model. For each cell-type-specific sub-population, we removed the single cells with <200 peaks and peaks without signals across all single cells. Moreover, additionally, cell types represented by fewer than 100 single cells were filtered out. These tissue/cell-type-specific count-based matrices were then utilized for the subsequent calling for enhancers. When relative fragment files were available, they were combined with the count-based matrices to enable the calling of SEs.

### 2.3 Characterization of single-cell typical enhancers and relative target genes

To identify single-cell typical enhancers from hundreds of tissue/cell-type-specific single cells, we improved a previously designed unsupervised method (Gao *et al.* 2022) by introducing a weighting system to assign quality scores to each single cell and combining all cells’ peak profiles to identify typical enhancers. In this approach, the ATAC peak profile of each single cell was treated as an independent dataset. Our method operates under the assumption that higher-quality datasets are more strongly associated with predicted enhancers, while lower-quality datasets have weaker associations. By comparing the similarities among all single-cell datasets, a relative quality score was assigned to each dataset. Traditionally, the scATAC-seq matrix is binarized to reflect the “open” or “closed” state of chromatin, based on the sparsity of the data and the conceptual framework of chromatin accessibility (Luo *et al.* 2024). However, a recent study demonstrated that modeling fragment counts, rather than binarizing the matrix, preserves quantitative regulatory information and improves the analysis of scATAC-seq data (Martens *et al.* 2024). To better evaluate the similarity between the datasets of any two single cells (e.g. A and B), we employed the Tanimoto coefficient to calculate their correlation. This approach allowed us to retain the quantitative nature of chromatin accessibility data, enhancing the accuracy of typical enhancer identification:

$$T_{AB}=\frac{A*B}{{\left|A\right|}^{2}|+{\left|B\right|}^{2}-A*B}=\frac{\sum_{i=1}^{n}A_{i}*B_{i}}{\sqrt{\sum_{i=1}^{n}{A_{i}}^{2}}+\sqrt{\sum_{i=1}^{n}{B_{i}}^{2}}-\sum_{i=1}^{n}A_{i}*B_{i}}$$

Within one tissue/cell-type-specific matrix containing n peaks and m cells, the $A_{i}$ and $B_{i}$ represent the fragment counts of peak i in cells A and B. To compute a weight for each cell A(i), we constructed a Tanimoto coefficient matrix to evaluate the similarity among all m cells as:

$$\left[\begin{array}{cc} \begin{array}{ccc} T_{A(1)A(1)} & \cdots & T_{A(1)A(i)}\\ \vdots & & \;\vdots \end{array} & \begin{array}{cc} \cdots & T_{A(1)A(m)}\\ & \;\vdots \; \end{array}\\ \begin{array}{ccc} T_{A(i)A(1)} & \cdots & T_{A(i)A(i)}\\ \;\vdots & & \vdots \\ T_{A(m)A(1)} & \cdots & T_{A(m)A(i)} \end{array} & \begin{array}{cc} \cdots & T_{A(i)A(m)}\\ & \vdots \\ \cdots & T_{A(m)A(m)} \end{array} \end{array}\right]$$

Then the weight of any single cell A(t)can be defined using the Tanimoto coefficient matrix as follows:

$$w_{A(t)}=\frac{\sum_{i=1}^{m}T_{A(t)A(i)}}{\sum_{i=1,k=1}^{m}T_{A(i)A(k)}}\;(i,k\in [1,m],i\neq t,i\neq k)$$

Next, for a cell type containing single cells, the consensus signal score of any peak i can be computed as the sum of each cell’s weight multiplied by its relative fragment count:

$$S_{\mathrm{combined}}(i)=\sum_{t=1}^{m}{{\mathrm{peak}}_{A(t)}(i)*w}_{A(t)}$$

where the ${\mathrm{peak}}_{A(t)}(i)$ means the fragment count of the peak i in the single cell A(t). Finally, all combined peaks undergo a quality control process based on four criteria: (i) each combined peak must have signals in at least one-third of all single cells within the cell type; (ii) peaks must not overlap with promoters or exons; (iii) to ensure the combined peak signal is not random, it must exceed the 95th percentile of signals from randomly shuffled peaks, computed by shuffling all peaks across the entire DNA sequence for each cell; and (iv) each combined peak must have a co-accessibility score of at least 0.1, as calculated by Cicero (Pliner *et al.* 2018), indicating a strong interaction with a promoter.

As a powerful predictor for cis-regulatory interactions, Cicero has been widely applied on scATAC-seq data to identify enhancer-promoter interactions at the whole DNA level (Cusanovich *et al.* 2018, Satpathy *et al.* 2019, Domcke *et al.* 2020, Zhang *et al.* 2021). In this study, we employed Cicero to identify the enhancer target promoter related gene for all tissue/cell types. However, due to the count-based sparsity of tissue- and cell-type-specific scATAC-seq matrices, accurately assessing co-accessibility scores among chromatin accessibility sites is challenging without continuous signal representation (Fang *et al.* 2021). To address this limitation, we utilized the latent semantic indexing (LSI) approach to transform the count matrix into a term frequency-inverse document frequency (TF-IDF) matrix with continuous values. Dimensionality reduction was subsequently performed using singular value decomposition (SVD) and uniform manifold approximation and projection (UMAP). For downstream analysis and two-dimensional visualization, dimensions 2–50 were selected from the reduced TF-IDF matrix. Using the Graphical LASSO algorithm, Cicero processed the transformed continuous matrix and UMAP coordinates to compute co-accessibility scores among chromatin accessibility sites within a defined distance threshold (e.g. 500 000 bp in human). This analysis identified a substantial number of enhancer–promoter connections. By applying a co-accessibility score cutoff of 0.1, we identified a total of 10 402 346 enhancer–promoter interactions across 1028 tissue and cell types in 13 species.

### 2.4 Identification of super-enhancer in single-cell level

In dbscATAC, we utilize the updated ROSE method (https://github.com/stjude/ROSE) to identify 213 835 single-cell SEs across 520 tissue/cell types in human, mouse, and fly. Most existing studies for calling SEs focus on H3K27ac ChIP-Seq data, which serves as a widely recognized marker for active enhancers and SEs (Khan and Zhang 2016, Wei *et al.* 2016, Jiang *et al.* 2019, Wang *et al.* 2023). Additionally, other key indicators of SEs, including P300, MED1, and BRD4 ChIP-Seq data, have been effectively employed to identify SEs (Chen *et al.* 2020, Hamamoto *et al.* 2023). Importantly, recent studies have demonstrated the feasibility of using bulk and even single-cell ATAC-seq data to call SEs, showing significant overlap with H3K27ac-identified SEs and validating their activities experimentally (Lee *et al.* 2021, Yates *et al.* 2021, Riegel *et al.* 2023). In this work, to achieve the identification of single-cell SEs, we leveraged the fragment file generated from scATAC-seq raw data and split it into cell-type-specific partitions to call SEs. If the scATAC-seq data was assigned with only the BAM file, the tool sinto (v0.10.1, https://timoast.github.io/sinto/basic_usage.html) could call it to create the necessary fragment files (Stuart *et al.* 2021). The process of ranking enhancers and identifying SEs from scATAC-seq BAM or fragment files involved six well-defined steps, ensuring accurate SE annotation.

Individual candidate enhancers were identified using chromatin markers derived from ATAC-seq data. First, each segmented cell-type-specific fragment file from scATAC-seq was sorted and converted into a cell-type-specific standard BAM file using bedtools and samtools (Quinlan and Hall 2010, Danecek *et al.* 2021). Next, macs2 (Zhang *et al.* 2008) was employed to call peaks, generating a narrowPeak file. The narrowPeak file was then transformed into a GFF file containing candidate enhancer regions using the Linux command awk. Finally, the transformed GFF file and the cell-type-specific standard BAM file were used as inputs for the ROSE algorithm to identify SEs.

We stitched the adjacent candidate enhancers located within a certain distance (typically 12.5 kb, 12.5 kb, 2 kb for human, mouse, and fly, respectively) together into larger enhancer regions, thereby forming candidate SEs. For a given set of enhancers $e_{1}$,$e_{2}$,…,$e_{n}$, the stitched enhancer (i.e. candidate super-enhancer) $S_{e}$ is represented as:

$$S_{e}=\overset{n}{\underset{i}{⋃}}e_{i}(\mathrm{distance}(e_{i},e_{i+1})\leq d$$

where d is the predefined stitching distance (e.g. 12.5 kb for human). Enhancers that are close to each other are merged into a single stitched candidate SE region.

For each stitched candidate SE $S_{e}$, the total enhancer signal is calculated by integrating the ATAC-seq signal across the entire region of the candidate SE. This signal often represents the level of ATAC-seq signal, integrated across the entire stitched region. If the ATAC-seq signal at a position t within an enhancer is given as Signal(t), then the total signal for the stitched enhancer is

$$\mathrm{Signal}(S_{e})=\int_{S_{e}}^{\;}\mathrm{Signal}(t)dt$$

Once the total signal is calculated for all stitched enhancers, the candidate SEs are ranked by their signal strength in ascending order. Given one cell type has n candidate SEs, The rank of any candidate super-enhancer can be calculated as:

$$R_{i}=\mathrm{rank}(\mathrm{Signal}(S_{e}(i)))\;(i\in [1,n],\mathrm{Signal}(S_{e}(1))\leq \mathrm{Signal}(S_{e}(2))\leq \ldots \leq \mathrm{Signal}(S_{e}(n)))$$

Then we plot the total enhancer signal with relative rank. The candidate SEs are categorized into “false SEs” and “true SEs” by finding a point of inflection on the rank plot. Mathematically, given the signal for candidate super-enhancer $S_{e}(i)$ and its rank $R_{i}$, then the slope between two consecutive points on the rank plot is:

$${\mathrm{Slope}}_{i}=\frac{\mathrm{Signal}(S_{e}(i))-\mathrm{Signal}(S_{e}(i+1))}{R_{i}-R_{i+1}}\;(i\in (1,n))$$

where $\mathrm{Signal}(S_{e}(i))$ represents the total ATAC-seq signal (fragment count) of the i-th ranked stitched enhancer, while $R_{i}$ means the Rank index (ascending order, $R_{i}$ = 1 for the weakest signal). The point at which the slope equals 1 and begins to steepen significantly is used to define true SEs. This inflection point serves as the threshold: enhancers above this threshold are classified as “true super-enhancers,” while those below it are considered “false super-enhancers.” Finally, the identified single-cell typical enhancers are used as a key filtering criterion, and only “true super-enhancers” that intersect with these typical enhancers are retained.

### 2.5 Annotation of tissue/cell-type-specific gene markers from scATAC-seq in 13 species

Similar to scRNA-seq, scATAC-seq can also be utilized to identify tissue/cell-type-specific gene markers by aggregating the accessibility signals across gene bodies and promoter regions (Granja *et al.* 2021, Stuart *et al.* 2021). We inferred the regulatory activity of genes by calculating the chromatin accessibility near the gene body regions (e.g. promoters and introns). Given a gene g that spans a genomic region from ${\mathrm{TSS}}_{g}$ (transcription start site) to ${\mathrm{TES}}_{g}$ (transcription end site), the total gene activity score can be calculated as:

$${\mathrm{Scor}e}_{g}=\sum_{i={\mathrm{TSS}}_{g}}^{{\mathrm{TES}}_{g}}\mathrm{Accessibility}(i)+\sum_{j={\mathrm{TSS}}_{g}-d}^{{\mathrm{TSS}}_{g}}\mathrm{Accessibility}(j)$$

where Accessibility(i),${\;\mathrm{TSS}}_{g}$, ${\mathrm{TES}}_{g}$, and d represent the chromatin accessibility signal at position i in the gene body, the transcription start site, transcription end site of gene g, and the distance upstream of the TSS (e.g. 2 kb) for capturing the promoter accessibility, respectively. This summation adds up the chromatin accessibility (e.g. ATAC-seq fragment counts) across the entire gene body and promoter regions.

For each scATAC-seq sample, the large peak matrix was transformed into a gene activity matrix using the described method. After normalizing this gene matrix, the “FindAllMarkers” function in Seurat was applied to identify tissue/cell-type-specific gene markers. To ensure the statistical significance of the identified differentially expressed genes, we set stringent thresholds: an adjusted *P*-value with cutoff as 1e−5, a minimum fraction of expressed cells in relative tissue/cell type as 0.3, and a log2 fold change threshold as 0.585.

### 2.6 Significance calculation of SE overlaps between dbscATAC and other databases

We validate the reliability of the SEs identified in dbscATAC with other well-known SE databases. To rigorously rule out random overlap, we performed hypergeometric tests for all overlap comparisons. We calculate the hypergeometric *P*-value as:

$$p=\frac{\left(\begin{array}{c} K\\ k \end{array})(\begin{array}{c} N-K\\ n-k \end{array}\right)}{\left(\begin{array}{c} N\\ n \end{array}\right)}$$

where the total number of possible SE regions (N) was defined as the union of all putative SEs from both databases (dbscATAC and the reference database) across the genome. K represents the total SEs in the reference database (e.g. dbSUBER: 325 cerebellum SEs). n equals the total SEs predicted by dbscATAC in the same tissue (e.g. dbscATAC: 312 cerebellum SEs). k means overlapping SEs between dbscATAC and the reference database. Especially, all SE coordinates were lifted to hg38 using liftOver to ensure compatibility. Regions failing coordinate conversion were excluded.

### 2.7 Implementation of dbscATAC

dbscATAC was constructed on the linux ubuntu 20.04.6 system and served as a robust web server using an environment configured with nginx (1.18.0)-php (7.4.3)-mySQL (6.0.11)-HTML5. Additionally, Perl (5.32.1) and Python (3.9.18) were also utilized to process large number of raw data efficiently before inputting into the website. Notably, we combined the HTML5 Canvas API with JavaScript to develop a genome browser for visualizing the distributions of the SEs, typical enhancers, gene markers, and single nucleotide polymorphisms (SNPs) within the DNA regions. A two-handle slider was integrated at the top of the canvas, enabling users to dynamically expand or contract the displayed genomic regions for detailed exploration. Moreover, we successfully developed a powerful online module for UMAP, Vlnplot, and heatmap plotting, and intuitively displaying the cell-type specificity of enhancers/gene markers or the differences among different cell types at the single-cell level. The platform also features several versatile analytic modules on the homepage and a hierarchical search function on the browse page, allowing users to seamlessly search for SEs, enhancers, and gene markers. Finally, dbscATAC is compatible with multiple operating systems, including Windows, Linux, and macOS, and is accessible through most modern web browsers (e.g. Internet Explorer, Google, Microsoft Edge, Safari, UC Browser, and Firefox).

## 3 Results

### 3.1 Resource statistics

dbscATAC annotated 213 835 SEs across 520 tissue/cell types in three species, as well as 347 484 gene markers, 13 470 526 enhancers, and 10 402 346 enhancer–promoter interactions across 1028 tissue/cell types in 13 species from 1 668 076 single cells at the single-cell resolution (Table 1). The identified results for all cell types were summarized and integrated separately for SEs gene markers, and enhancers, encompassing numerous diseases and covering most animal and plant tissues.

To achieve these annotations, we employed the ROSE method, an improved unsupervised learning approach we developed, the chromatin accessibility signal aggregation method from Signac, and cis-regulatory network analysis based on Cicero. These methodologies were used to identify single-cell SEs, enhancers, gene markers, and enhancer-promoter interactions, respectively (Fig. 1). Most tissue/cell types demonstrated rich chromatin accessibility information, with each single cell containing an average of at least 3000 peaks (Table 1, available as supplementary data at *Bioinformatics* online). We only predicted the SEs in human, mouse, and fly (Table 2, available as supplementary data at *Bioinformatics* online). The identification of gene markers was conducted using scATAC-seq projects with at least three annotated cell types (Table 3, available as supplementary data at *Bioinformatics* online). The single-cell consensus enhancers were determined using data from at least 100 single-cell datasets to ensure robustness and reliability (Table 4, available as supplementary data at *Bioinformatics* online). For other species without SEs, fragment files or raw sequencing data were unavailable, and genome annotations remain insufficiently resolved for robust SE identification. We will extend SE predictions to additional species in future updates as these resources become available.

### 3.2 SE validation through comparison with other SE databases

**Figure 2.**
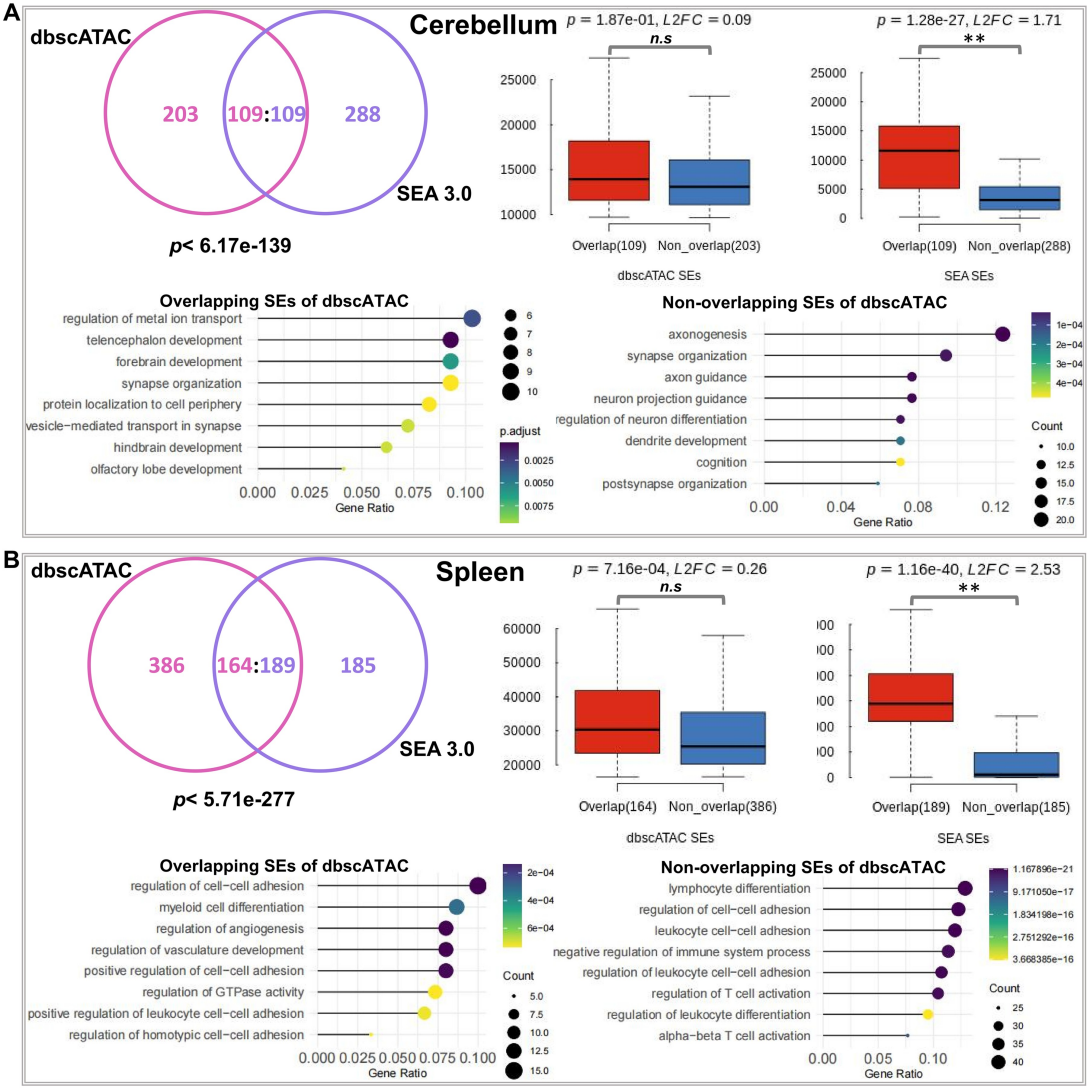
SE annotation validated by other SE resources. (A) Comparison with SEA3.0 in cerebellum. (B) Comparison with SEA3.0 in spleen. The *P* values were calculated using the hypergeometric test in Venn diagram and Student’s *t*-test in boxplot. **P < 1e−10 and L2FC > 1; n.s. not significant.

**Figure 3.**
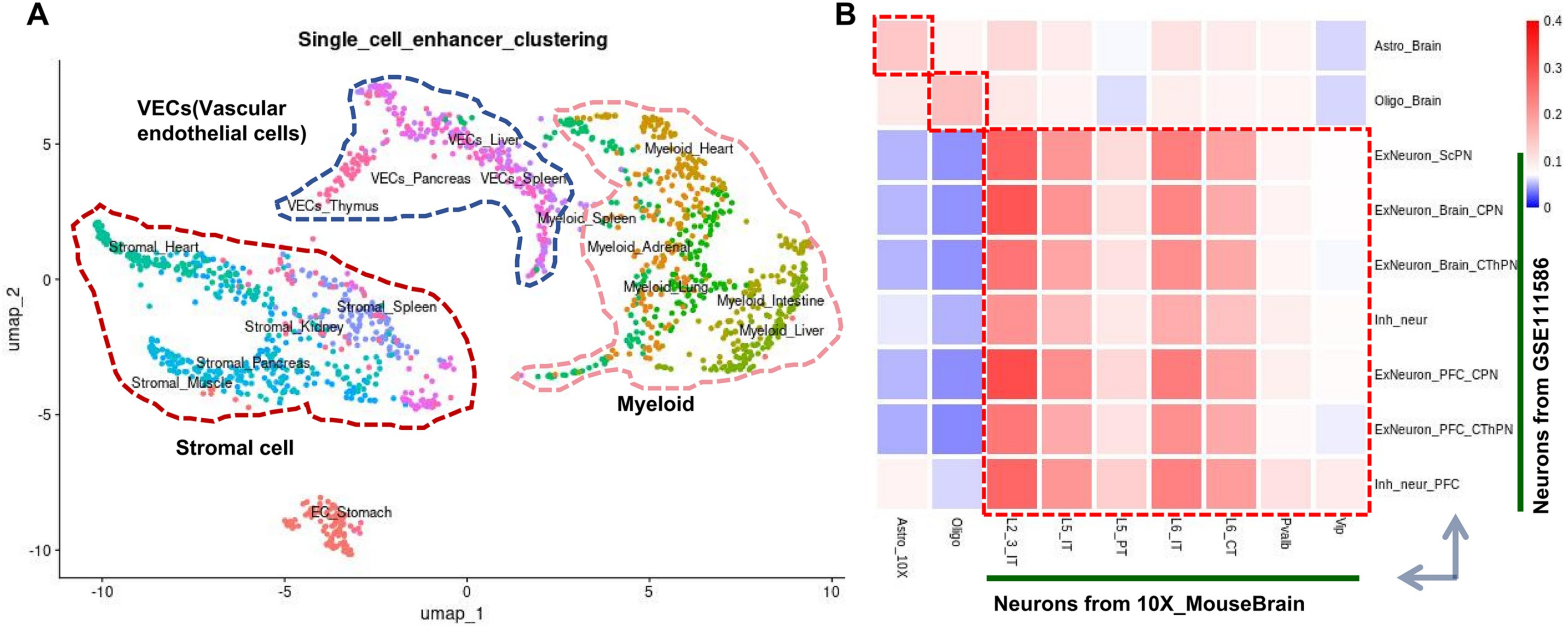
Robustness and consistency of enhancer annotation among similar tissue/cell types. (A) Different batches from the source GSE149683 displayed limited batch effects. (B) Similar cell types from two projects with significant batch effects exhibit good similarity of enhancer annotations.

**Figure 4.**
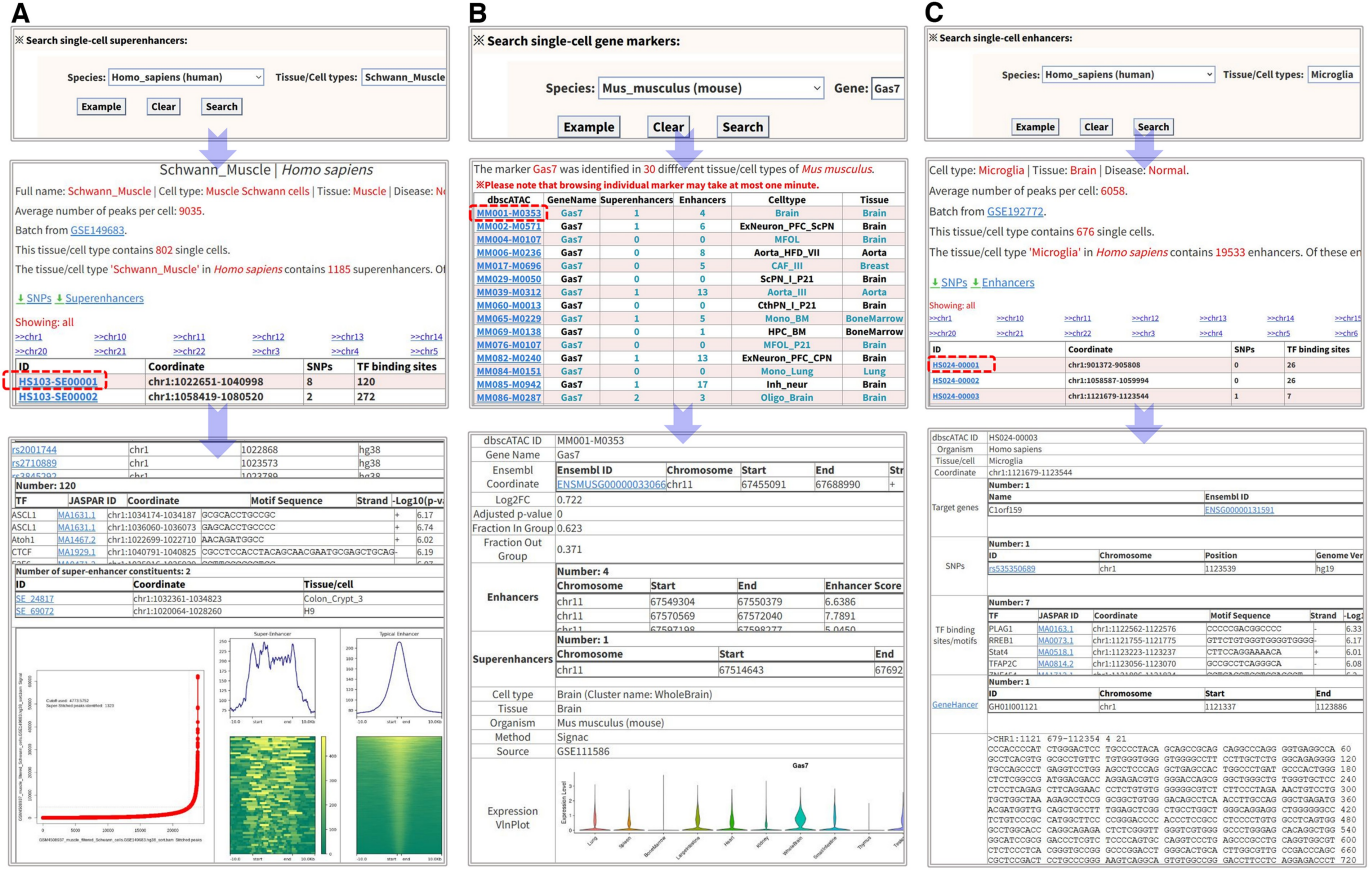
Simple search with three web-based tools. (A) Search tissue/cell-type-specific single-cell SEs by the selected tissue/cell type. (B) Retrieve tissue/cell-type-specific gene markers by a given species. (C) Search tissue/cell-type-specific single-cell enhancers by a DNA region of interest.

To validate the reliability of the SEs identified in dbscATAC, we compared their similarities with those from other well-known SE databases, such as dbSUPER, SEA3.0, and SEdb 2.0 (Khan and Zhang 2016, Chen *et al.* 2020, Wang *et al.* 2023). Our results demonstrate that 34.94% (109/312) of dbscATAC SEs overlap with 27.46% (109/397) of SEA3.0 SEs in cerebellum, while 29.82% (164/550) of dbscATAC SEs overlap with 50.53% (189/374) of SEA3.0 SEs in spleen. These overlaps exhibit statistical significance (cerebellum: *P *< 6.17e−139; spleen: *P *< 5.71e−277, hypergeometric test; Fig. 2). Similarly, comparisons with dbSUPER revealed significant overlaps (cerebellum: *P *< 3.54e−184; spleen: *P *= 0; Fig. 2, available as supplementary data at *Bioinformatics* online). Interestingly, we compared signals between non-overlapping SEs and overlapping SEs, and observed non-significant difference in dbscATAC against significant difference in SEA 3.0. For example in cerebellum, overlapping SEs displayed much higher signals than non-overlapping SEs with *P *= 1.28e−27 and log base 2 fold change (L2FC) = 1.71 in SEA 3.0, while no significant difference (*P *= 1.87e−01 and L2FC = 0.09) were observed in dbscATAC. More, the GO analysis showed that SE-associated genes in overlapping between dbscATAC and SEA3.0 likely represent core regulatory elements, whereas genes in non-overlapping may mark cell-state-specific activity (e.g. axon/neuron; Fig. 2A). Similarly in spleen, the signal comparisons between non-overlapping SEs and overlapping SEs revealed non-significant (*P *= 7.16e−04 and L2FC = 0.26) and significant (*P *= 1.16e−40 and L2FC = 2.53) differences in dbscATAC and SEA3.0, respectively. Comparing with overlapping SEs, non-overlapping SEs showed enrichment for pathways specific to rare cell types (e.g. lymphocyte differentiation and T cell activation), indicating their biological relevance (Fig. 2B). Significant overlaps between dbscATAC and SEA 3.0 were also observed with *P *< 2.10e−47, *P *< 5.01e−285, and *P *< 2.07e−34 in heart, kidney, and lung, respectively (Fig. 3, available as supplementary data at *Bioinformatics* online). Additionally in human, the overlapping analysis of SEs between dbscATAC and SEdb 2.0 also displayed statistically significant overlaps in shared thymus, placenta, muscle, and bone marrow tissues with *P *< 1.39e−196, *P *< 1.07e−101, *P *< 2.13e−30, and *P *< 7.41e−20, respectively (Fig. 4, available as supplementary data at *Bioinformatics* online). These results implied that the partial overlap between dbscATAC and existing SE databases arises from technical (data type) and biological (cell-type specificity) factors. Rather than a limitation, this highlights the unique value of single-cell resolution in uncovering previously masked regulatory diversity.

### 3.3 Robust and consistent annotation of enhancers among similar tissue/cell types with limited batch effect

To minimize batch effects, we avoided integrating different scATAC-seq projects but instead called super-enhancers and enhancers separately from the cell types within each individual project. We observed two interesting patterns. First, different batches from the same source tended to exhibit minimal batch effects, likely due to effective batch control and the use of a common peak set in the processing of scATAC-seq data. For example, the source from GSE149683, which mapped an atlas of human fetal tissues across three different batches at the single-cell level (Zhang *et al.* 2021), showed that similar tissue/cell types from different batches (i.e. tissues) could still be clustered together in the analysis (Fig. 3A). Second, while scATAC-seq data from different projects often exhibited substantial batch effects, the aggregated enhancer annotations for each cluster remained similar. For instance, we examined astrocytes, oligodendrocytes, and neurons in the mouse brain from two projects: “10X_MouseBrain” (https://stuartlab.org/signac/articles/mouse_brain_vignette) and GSE111586 (Cusanovich *et al.* 2018). Although these cell types showed significant batch effects at the single-cell level between the two projects, the similarity of enhancer annotations, as measured by Jaccard coefficients, was still high (Fig. 3B). This suggests that the enhancer annotations derived from scATAC-seq data across different projects are robust and consistent in dbscATAC.

### 3.4 Resource search with several analytic modules

In dbscATAC, we designed three simple modules and three advanced modules for search of single-cell SEs, enhancers, and gene markers in a user-friendly way (Fig. 4). These modules allow users to perform straightforward searches and visualize tissue/cell-type-specific SEs, enhancers, and gene markers with three web-based tools: (i) Search single-cell super-enhancers (Fig. 4A). (ii) Search single-cell gene markers (Fig. 4B). (iii) Search single-cell enhancers (Fig. 4C).

**Figure 5.**
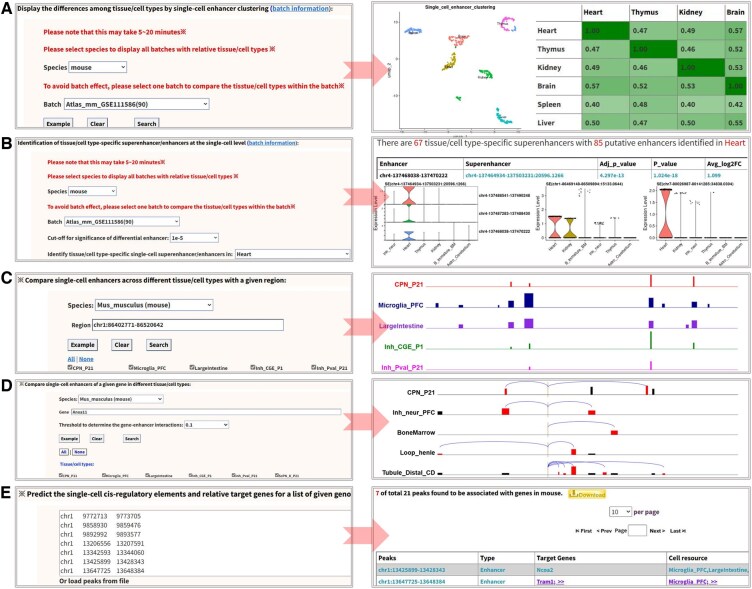
Visualization of differences among tissue/cell-type-specific single-cell enhancers. (A) Analyze the differences of scATAC-seq data among different tissue/cell types by clustering. (B) Identify the tissue/cell-type-specific enhancers at the single-cell level. (C) Search and compare the single-cell enhancer distributions across selected tissue/cell types with a given region. (D) Given a gene, find and compare its enhancers across selected tissue/cell types. (E) Predict the enhancer or promoter properties and even their target genes for the input peak regions.

To enable complex analyses, we have implemented five advanced search options that facilitate users perform online comparative analyses on single-cell enhancers across different cell types, and visualize the corresponding results within an integrated genome browser (Fig. 5). To minimize batch effects as much as possible, we assigned each tissue/cell type to a specific batch and conducted comparisons only among different tissue/cell types within the same batch. Based on this approach, all the tissue/cell types were categorized as 7, 3, 1, 1, 1, 1, 1, 1, 1, 1, and 1 batches for human, mouse, fly, cynomolgus, marmoset, chimp, rhesus, zebrafish, arabidopsis, rice, and maize, respectively. Notably, two powerful online modules were designed to exhibit the differences among selected tissue/cell types and the tissue/cell type specificity of single-cell enhancers intuitively (Fig. 5A and B).

### 3.5 Browse of SEs, enhancers, and gene markers

**Figure 6.**
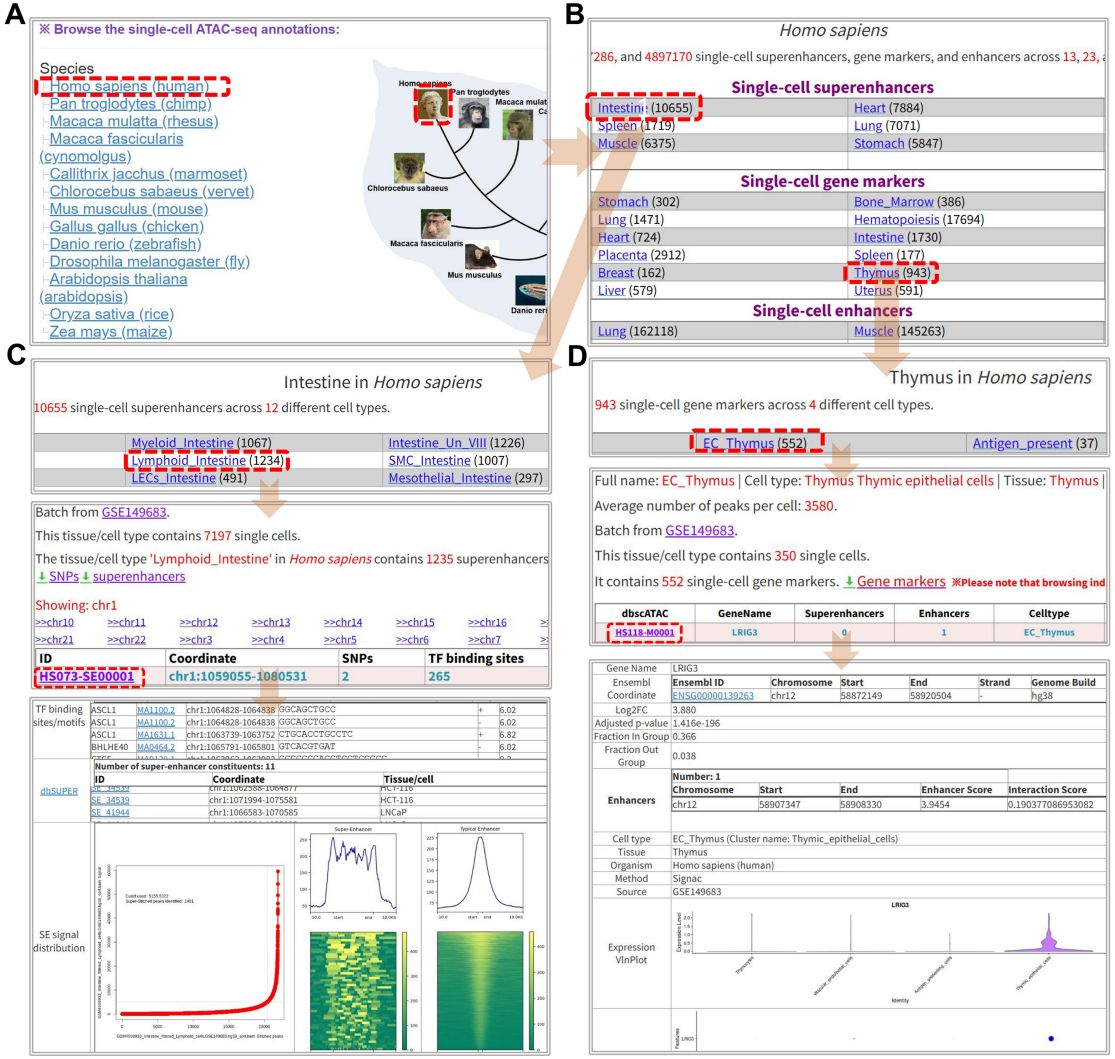
Browse of SEs, gene markers, and enhancers. (A) Select one species of interest by clicking its name or image. (B) Choose a specific tissue (e.g. Brain) within the selected species to view relative SEs and gene markers. (C) List all SE related cell types in the selected tissue. Select a specific cell type to view its SEs and access comprehensive details for any chosen SE. (D) Summarize the cell types within the selected tissue under “Single-cell gene markers.” Choose a specific cell type to view its gene markers and explore detailed information for any chosen marker.

A dedicated page was designed with a hierarchical browsing structure for users to access any single-cell SEs, gene markers and enhancers by species, tissues, and cell types. Additionally, we annotate SEs/enhancers with related Genome-Wide Association Studies (GWAS) SNPs (Buniello *et al.* 2019), TF binding sites from JASPAR (Fornes *et al.* 2020), enhancer-associated diseases (Wang *et al.* 2018), and DNA base sequences. For single-cell gene markers, their annotations include links to Ensembl (Flicek *et al.* 2012), associated SEs or enhancers, and online visualizations of gene expression levels derived from scATAC-seq clustering, generated using Seurat (Hao *et al.* 2024). Users can easily browse tissue/cell types, and their associated SEs, gene markers, and enhancers by clicking on the species name or image of interest (Fig. 6).

## 4 Conclusion and discussion

dbscATAC is the first resource to annotate SEs and gene markers based on scATAC-seq. It includes 213 835 SEs, 347 484 gene markers, 13 470 526 enhancers and 10 402 346 enhancer–gene interactions derived from 1 668 076 single cells across 1028 tissue/cell types in 13 species. Additionally, several useful analytic tools are designed online in the website. These make dbscATAC be the most comprehensive resource for single-cell SEs, gene markers, and enhancers.

For dbscATAC, it relied on single-cell ATAC-seq data, which captured cell-type-specific chromatin accessibility at single-cell resolution. The SE-associated genes in some cell types are tied to cell-type-specific functions and identity pathways (Fig. 5, available as supplementary data at *Bioinformatics* online). While this enabled discovery of subpopulation-specific SEs, the inherent sparsity of scATAC-seq data may limit detection of low-signal SEs (e.g. low signals of non-overlapping SEs of SEA 3.0; Fig. 2) that are only active in rare cell subsets. However, for dbSUPER/SEA3.0, they were built on bulk ChIP-seq data (e.g. H3K27ac/MED1/SOX2), which averages signals across cell populations. This approach favors SEs active across broad cell types but may obscure SEs unique to minor sub-populations. So the partial overlap between them reflected complementary strengths. Bulk methods identify conserved SEs across tissues, while scATAC-seq reveals subpopulation-specific SEs. The partial overlap between dbscATAC and existing databases might initially appear limited, its statistical significance supports biological relevance rather than random chance.

A recent study highlighted that SEs are composed of classical enhancers and facilitators. Unlike classical enhancers, facilitators did not have intrinsic enhancer activity and are functionally distinct. However, their presence is critical as classical enhancers exhibit reduced activity and diminished enhancer–gene interactions in their absence, failing to fully activate their regulated genes (Blayney *et al.* 2023). To better understand the nature of SEs, future efforts will involve developing a novel machine learning method to distinguish classical enhancers from facilitators within scATAC-seq peaks.

As a constantly evolving resource, dbscATAC will be regularly updated with newly available datasets. In the next version, additional datasets and features are planned to further enhance its functionality and comprehensiveness.

## Supplementary Material

btaf364_Supplementary_Data

## Data Availability

All the single-cell chromatin accessibility annotation data could be downloaded in http://singlecelldb.com/dbscATAC/download.php. And the source code of dbscATAC for prediction of SEs, enhancers, and gene markers are available at https://github.com/EvansGao/dbscATAC.
