# Supplementary material for: dbscATAC: a resource of single-cell super-enhancers/enhancers and gene markers derived from scATAC-seq data
Source: Bioinformatics. 2025 Jun 23;41(7):btaf364. doi: 10.1093/bioinformatics/btaf364 (PMC12237509; doi:10.1093/bioinformatics/btaf364)
Supplement: btaf364_Supplementary_Data [file btaf364_supplementary_data.zip › Supplementary_data.docx]

**Supplementary Data**

dbscATAC: a resource of single-cell super-enhancers/enhancers and gene markers derived from scATAC-seq data

**Yingmei Li ^1#^, Shahid Ullah ^2#^, Yumei Xian ^1,3#^, Yazhou Sun ^1,3^, Zilong Zheng ^1^, Xiaoyu Ma ^1,3^, Ming Shi^3^, Changlin Zhang ^4^, Tian Li ^4^, Leli Zeng ^5^, Jie Chen ^6^, Yubin YB Deng ^3^, Fuxin Wei ^7,8^* and Tianshun Gao ^1,3,8^***

^1^Big Data Center, The Seventh Affiliated Hospital of Sun Yat-sen University, Shenzhen 518107, China.

^2^S-Khan Lab Mardan, Khyber Pakhtunkhwa, Pakistan.

^3^Scientific Research Center, The Seventh Affiliated Hospital of Sun Yat-sen University, Shenzhen 518107, P.R. China.

^4^Department of Gynecology, The Seventh Affiliated Hospital of Sun Yat-sen University, Shenzhen 518107, China

^5^Digestive Diseases Center, Guangdong Provincial Key Laboratory of Digestive Cancer Research, Scientific Research Center, The Biobank, The Seventh Affiliated Hospital of Sun Yat-Sen University, Shenzhen 518107, China

^6^Department of General Surgery, Shanghai Children’s Medical Center, Shanghai Jiao Tong School of Medicine, Shanghai 200127, China

^7^Department of Orthopedic Surgery,The Seventh Affiliated Hospital of Sun Yat-sen University, Shenzhen, 518107, China

^8^Shenzhen Key Laboratory of Bone Tissue Repair and Translational Research. Shenzhen, 518107, China

#These authors contributed equally to this work

***** Corresponding authors


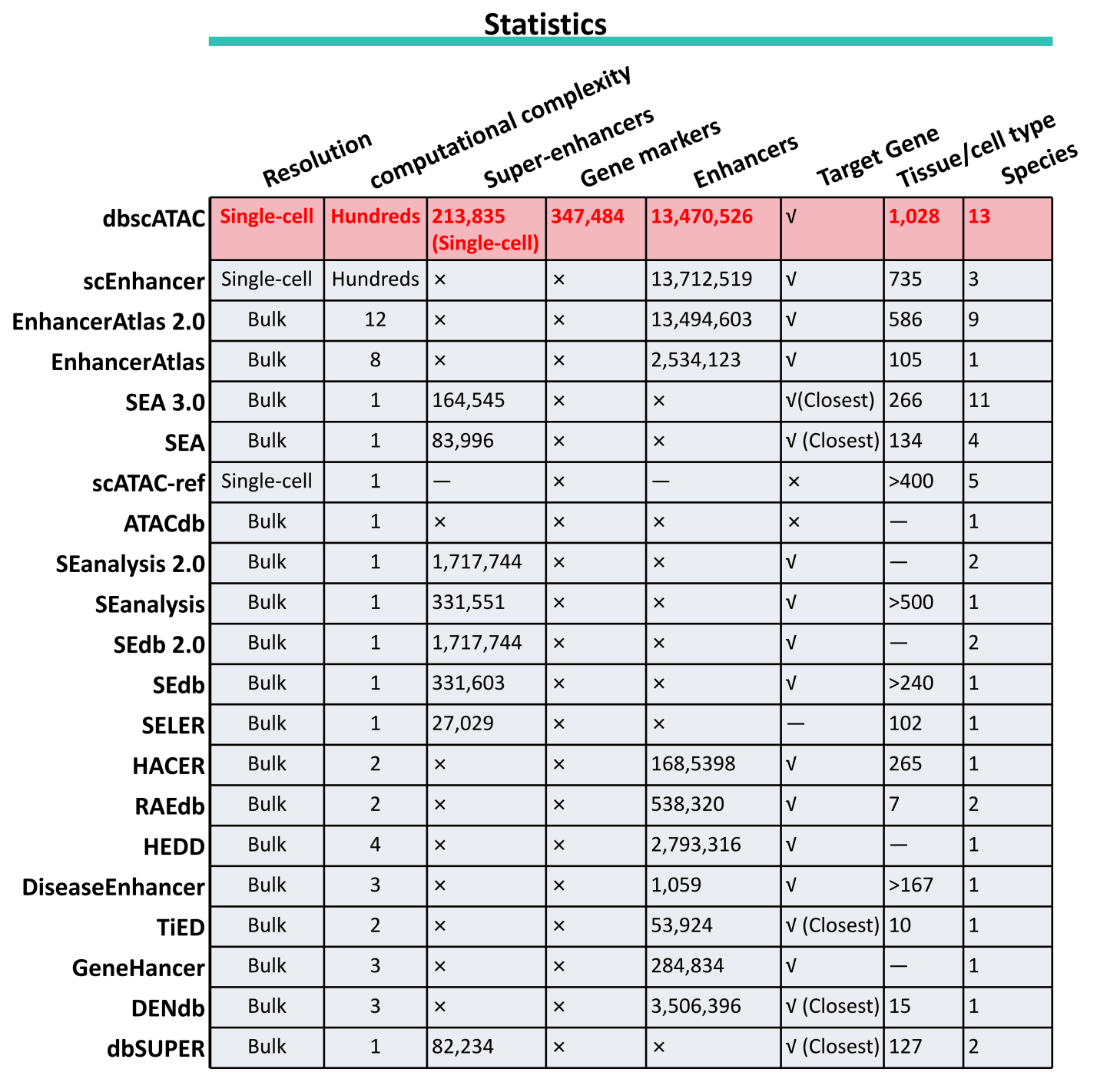


**Supplementary Figure S1.** Comparing dbscATAC with other databases. Here “computation complexity” means the number of cell type-specific datasets (e.g. an ATAC peak atlas per single cell) within one calculation for identification of cell type-specific super-enhancers or enhancers. Some resources set the “closest” gene as the enhancer target gene, while dbscATAC and scEnhancer utilized the deep learning tool Cicero to calculate the enhancer interacted gene promoter.


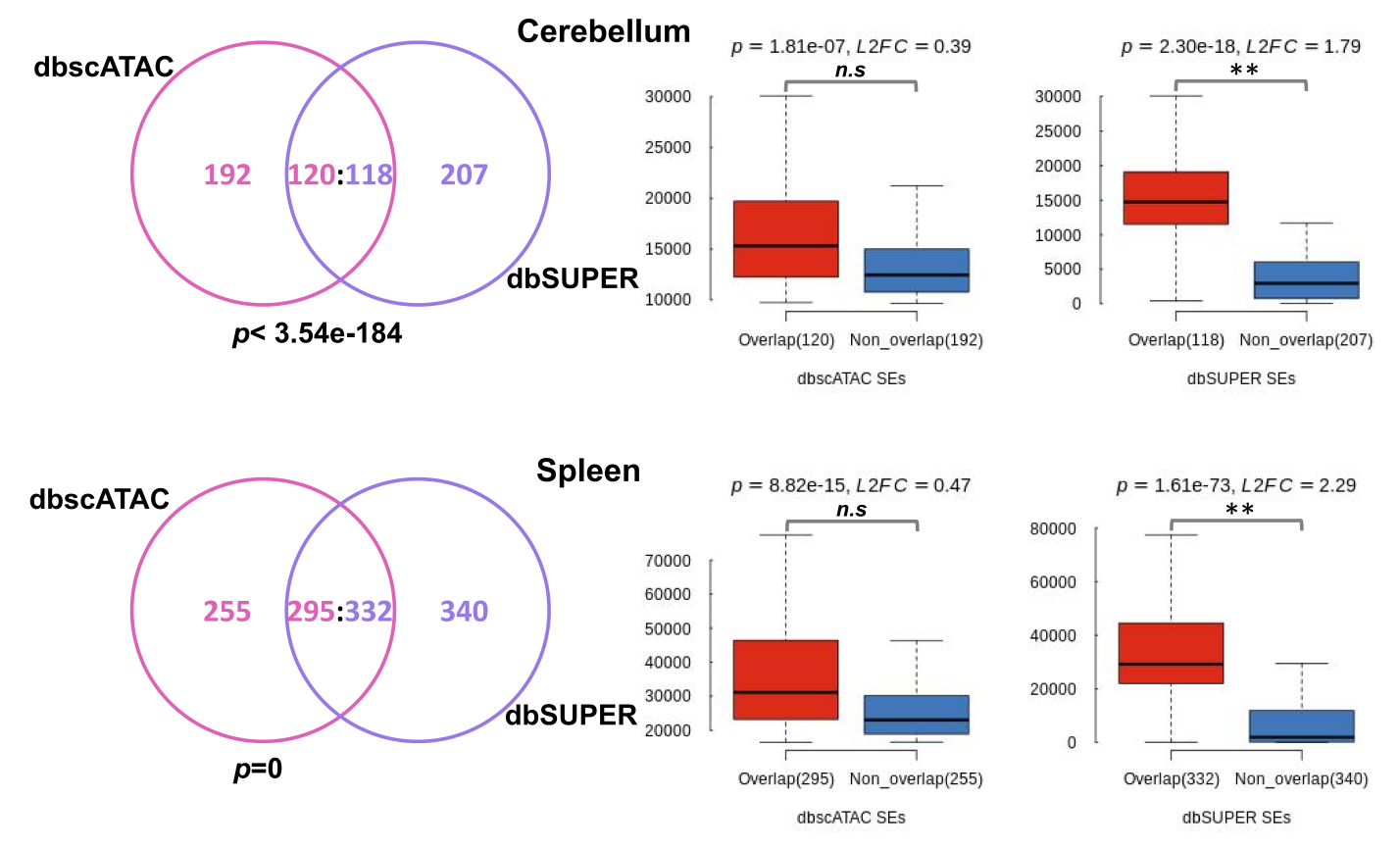


**Supplementary Figure S2.** comparisons with dbSUPER revealed significant overlaps (cerebellum: p< 3.54e-184; spleen: p=0).


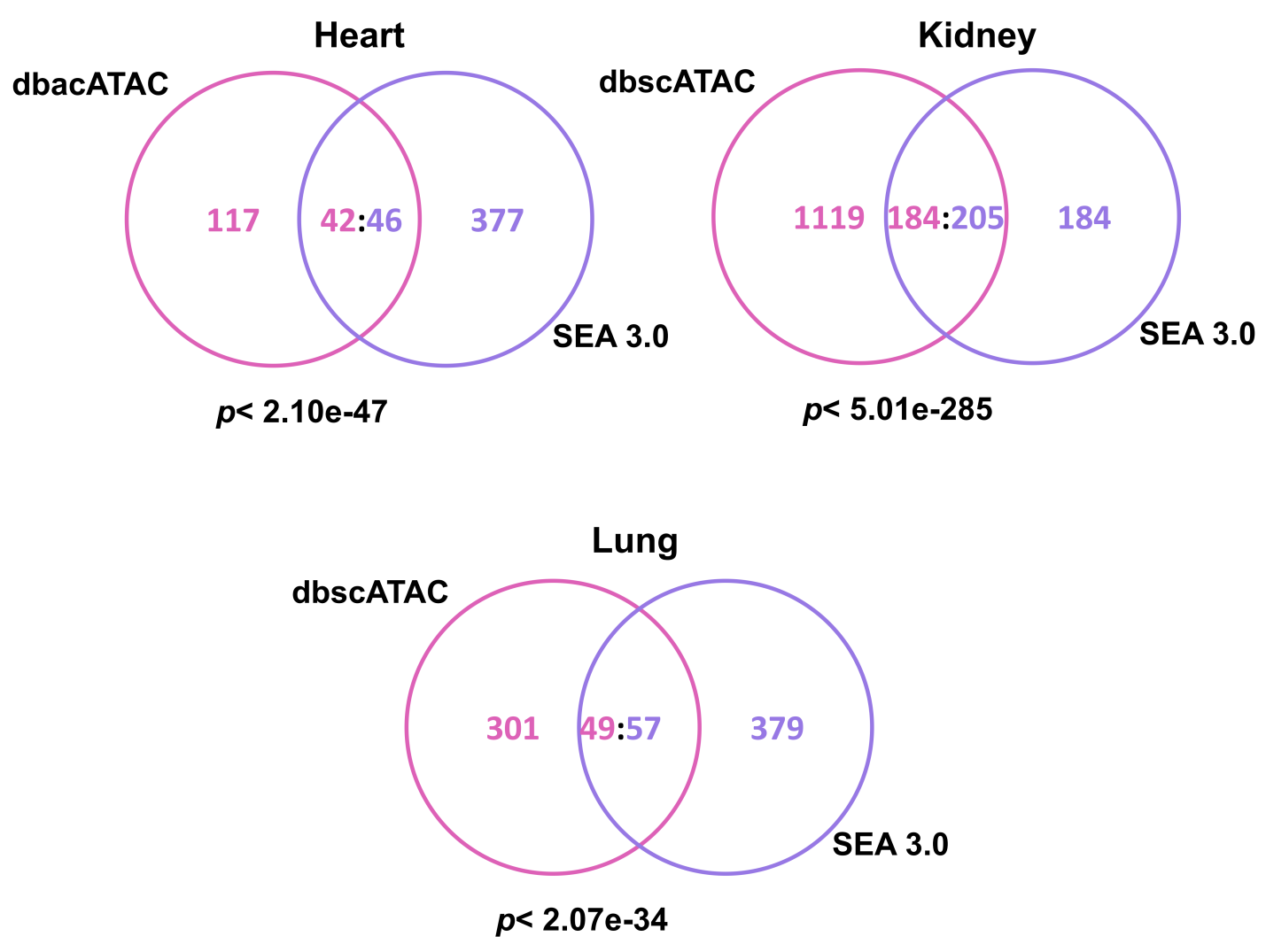


**Supplementary Figure S3.** Significant overlaps between dbscATAC and SEA 3.0 were also observed with p< 2.10e-47, p< 5.01e-285, and p< 2.07e-34 in heart, Kidney, and lung, respectively.


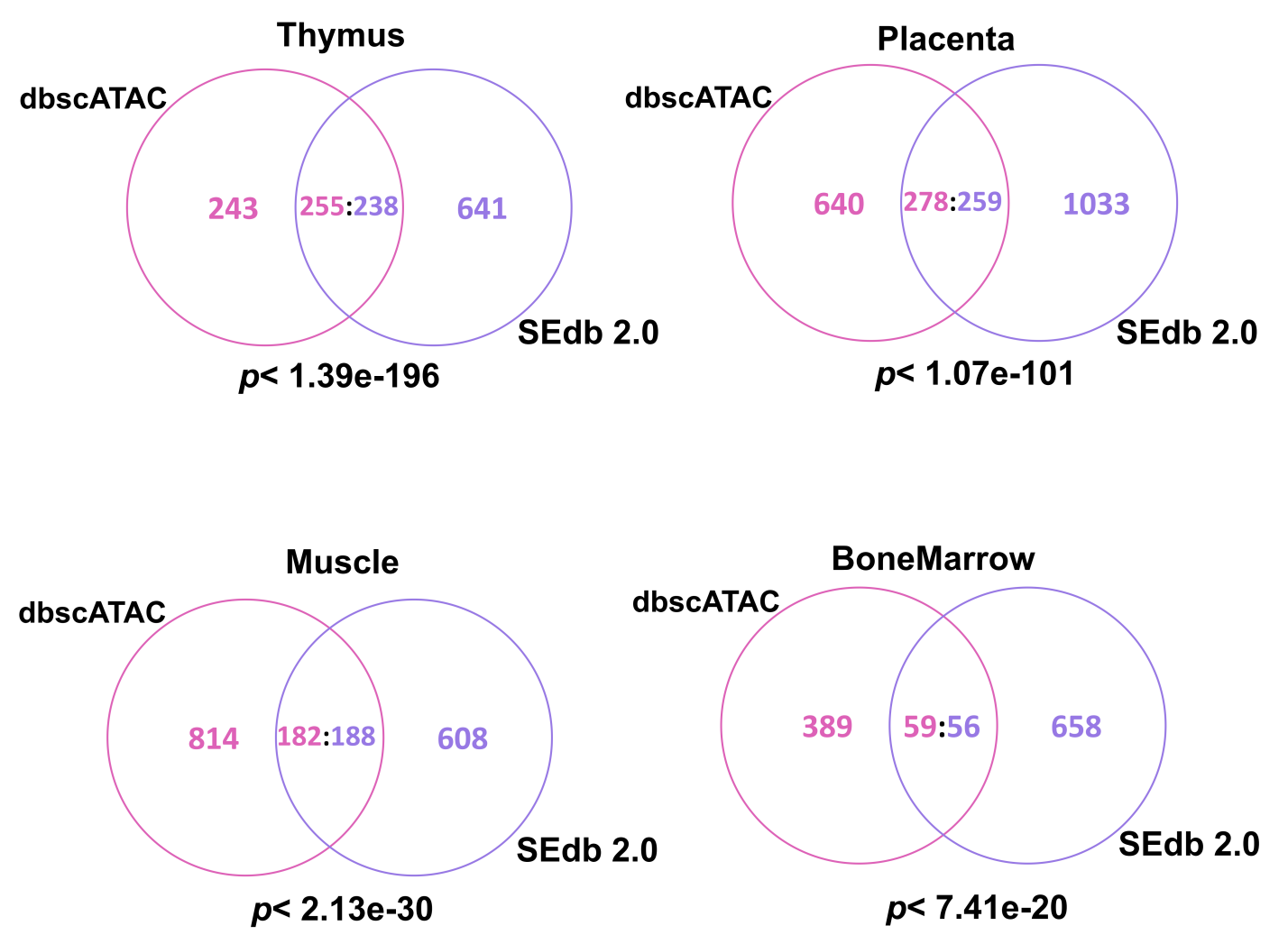


**Supplementary Figure S4.** In human, the overlapping analysis of SEs between dbscATAC and SEdb 2.0 displayed statistically significant overlaps in shared thymus, placenta, muscle, and bone marrow tissues with p< 1.39e-196, p< 1.07e-101, p< 2.13e-30, and p< 7.41e-20, respectively.


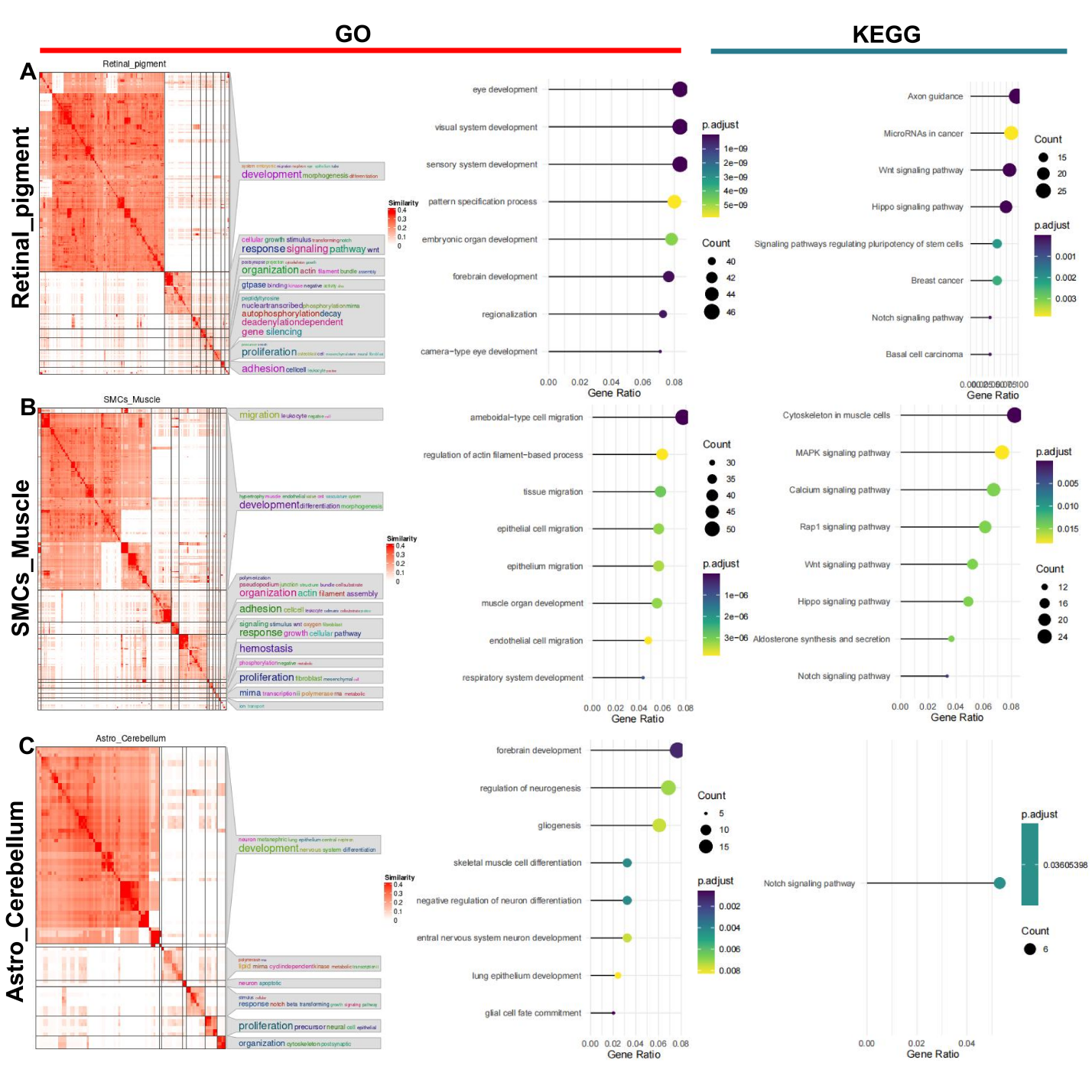


**Supplementary Figure S5. T**hree specific cell types, Retinal_pigment, SMCs_Muscle (Smooth muscle cells of muscle), and Astro_cerebellum (astrocyte of cerebellum) were performed for GO and KEGG enrichment analyses. For Retinal_pigment, SE-associated genes significantly enriched eye-related developments. The top three GO terms included eye development (GO:0001654, p.adjust=1.67e-12), visual system development (GO:0150063, p.adjust=1.67e-12), and sensory system development (GO:0048880, p.adjust=1.99e-12), while the KEGG results displayed significant retinal related axon guidance (hsa04360, p.adjust=2.60e-8), Wnt signaling pathway (hsa04310, p.adjust=3.16e-6) and Hippo signaling pathway (hsa04390, p.adjust=8.27e-6). For SMCs_Muscle, SE-associated genes mainly enriched muscle related development or function, including muscle organ development (GO:0007517,p.adjust=3.18e-6), regulation of actin filament-based process (GO:0032970, p.adjust=3.81e-6), and cytoskeleton in muscle cells (hsa04820, mostly significant in KEGG, p.adjust=6.41e-5). For Astro_cerebellum, SE-associated genes mainly enriched for glial cell fate commitment (GO:0021781, p.adjust=5.48e-4), forebrain development (GO:0030900, p.adjust=1.29e-3), regulation of neurogenesis (GO:0050767, p.adjust=7.07e-3), and gliogenesis (GO:0042063, p.adjust=7.57e-3).

**Supplementary Table S1.** Summary of all data in each tissue/cell type.

**Supplementary Table S2.** Summary of single-cell super-enhancers in all tissue/cell types.

**Supplementary Table S3.** Summary of single-cell gene markers in all tissue/cell types.

**Supplementary Table S4.** Summary of single-cell enhancers in all tissue/cell types.
